# Factors influencing happiness and depression in high-risk pregnant women: a cross-sectional study using the ecological systems approach

**DOI:** 10.4069/whn.2024.09.10

**Published:** 2024-09-30

**Authors:** Hyunkyung Choi

**Affiliations:** College of Nursing & Research Institute of Nursing Innovation, Kyungpook National University, Daegu, Korea

**Keywords:** Depression, Ecology, Happiness, High-risk pregnancy

## Abstract

**Purpose:**

The increasing number of high-risk pregnancies has led to a greater emphasis on psychological well-being in nursing care. However, reducing depression does not automatically equate to increasing happiness. This study aimed to systematically examine the factors influencing happiness and depression among high-risk pregnant women in South Korea.

**Methods:**

This correlational, cross-sectional study was based on the ecological systems theory. In total, 152 high-risk pregnant women completed a self-report survey questionnaire available online or offline. Data were analyzed using hierarchical regression analysis.

**Results:**

The first model (individual system) identified pregnancy stress and mindfulness as significant factors influencing both happiness and depression. The second model (microsystem) identified medical status at the time of the survey, maternal-fetal interaction, marital intimacy, and social support as additional significant factors influencing either happiness or depression. In the third model (mesosystem), maternal-fetal interaction and paternal-fetal attachment were no longer identified as significant factors. Although the fourth model (exosystem) did not identify community service as a significant factor, individual (pregnancy stress, mindfulness) and microsystem (marital intimacy) factors were found to influence happiness and depression. Medical status at the time of survey and social support were additional factors that influenced happiness, but not depression. These factors explained 51.2% and 55.5% of the variance in happiness and depression, respectively, among high-risk pregnant women.

**Conclusion:**

Different factors at the individual and microsystem levels affected happiness and depression among high-risk pregnant women. Hence, efforts to reduce depression among these women should be accompanied by efforts to actively promote happiness.

## Introduction

High-risk pregnancy involves risk factors that may endanger the health and life of both the pregnant woman and her fetus during pregnancy or childbirth [1]. In Korea, 95 such risk factors affecting pregnancy outcomes have been systematically classified into four categories: obstetric risk factors (e.g., history of preeclampsia), medical risk factors (e.g., diabetes mellitus), physical risk factors (e.g., underweight), and risk factors related to the current pregnancy (e.g., twin pregnancy) [1]. From 2006 to 2016, the number of pregnant women hospitalized for the eight most common diseases associated with high-risk pregnancy—namely, preterm labor, premature rupture of membranes, placenta previa, postpartum hemorrhage, pregnancy-induced hypertension, amniotic fluid and amniotic membrane disease, cervical incompetence, and gestational diabetes—increased by 3.5 times [2]. Assisted reproductive technology has led to higher pregnancy rates among women aged 35 years and older, subsequently increasing the incidence of high-risk pregnancies due to a rise in twin pregnancies [3].

Depression is a serious medical illness that negatively impacts an individual’s feelings, thoughts, and behaviors [4]. Research has found a strong link between experiencing depression during pregnancy and the development of postpartum depression [5]. High-risk pregnant women have higher levels of stress, anxiety, and prenatal depression than low-risk pregnant women [6]. Anxiety and stress have been identified as significant predictors of depressive symptoms during pregnancy [7]. A significant proportion of pregnant women hospitalized for preterm labor have been found to experience self-perceived burden and early postpartum depression [8]. Factors such as low self-esteem (individual) and low marital satisfaction and social support (environmental) have been associated with depression in pregnant women [5].

Meanwhile, happiness is a subjective judgment of an individual’s experiences shaped by personal and environmental factors that promote a more positive interpretation of those experiences [9,10]. Pregnant women who are happy tend to experience less stress, and high levels of happiness in this group have been positively associated with the release of hormones that support fetal growth and development [11]. Several factors have been identified as influencing maternal happiness, including individual elements like personality and pregnancy-related stress, as well as environmental aspects such as marital satisfaction and community services [12].

Previous studies have primarily focused on understanding and mitigating depression related to pregnancy [13]. Additionally, research has often concentrated on negative aspects such as anxiety and depression, particularly in high-risk pregnant women [7]. Happiness is an emotion, while depression is a medical condition that adversely impacts feelings, thoughts, and behaviors [4,9]. Happiness is not merely the absence of depression, nor is it simply being emotionally positive. Therefore, high-risk pregnant women frequently experience contradictory emotions; they can feel happy despite their anxiety and fear [14]. Likewise, resolving depression does not automatically result in happiness. Therefore, an integrated approach that promotes happiness in pregnant women is necessary, alongside efforts to reduce depression. To systematically identify factors that may influence happiness and depression in high-risk pregnancies, it is crucial to consider not only individual factors but also the environmental factors surrounding these pregnancies. Previous studies have applied ecological systems theory to explore factors affecting happiness and depression across various populations, including pregnant women [12].

**Figure 1.**
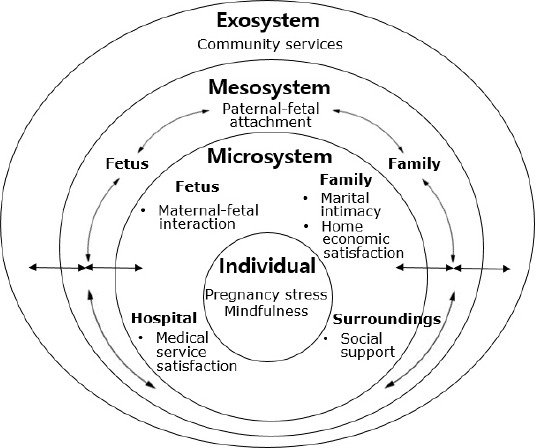
Conceptual framework for the study of happiness and depression in women with high-risk pregnancies using an ecological systems approach

According to Bronfenbrenner [15], humans cannot think in isolation from their environment and change as they interact with it, rather than existing independently. Similarly, the happiness and depression of pregnant women are influenced by their surroundings and manifest through these interactions [5,12]. The ecological systems theory views an individual who is pregnant as an organism, and previous studies have utilized the concept of pregnancy stress to explain pregnant women’s happiness [12] and prenatal depression [7,16]. Mindfulness is the awareness of one’s inner state and surroundings, and mindfulness practice can be used as a strategy to increase happiness [17]; furthermore, mindfulness-based programs have been shown to reduce prenatal depression [18]. Thus, pregnancy stress and mindfulness are individual system variables that may influence happiness and depression in high-risk pregnant women. The microsystem encompasses the environment with which a pregnant woman directly interacts, including the fetus, family, hospital, and support system. These elements have been shown to impact high-risk pregnant women according to previous research. Specifically, maternal-fetal interaction had been positively associated with maternal happiness [12] and negatively associated with postpartum depression [19]. Marital intimacy or marital satisfaction has been identified as a significant factor affecting maternal happiness and depression in pregnant women [12,20], whereas satisfaction with the family’s economic status and medical services has shown a significant association with happiness [12]. Given that high-risk pregnant women typically have more prenatal visits than their low-risk counterparts, their satisfaction with hospital services is likely to influence their happiness and/or depression. Additionally, support from family and friends has been positively associated with maternal happiness, whereas social support has been negatively associated with prenatal depression [12,16]. Mesosystem variables involve interactions between microsystem elements. For example, a pregnant woman’s perception of her spouse’s interaction with her fetus is an environmental factor that can influence her happiness and depression. In the context of increasing spousal participation during pregnancy, examining how paternal-fetal attachment affects pregnant women is necessary [21]. Although the exosystem does not directly influence the pregnant woman, it can affect the microsystem environments with which she closely interacts. Satisfaction with community services for pregnant women has been found to significantly influence maternal happiness [12]. Specifically, satisfaction with community services for high-risk pregnant women, who may require access to various healthcare facilities, is considered an environmental factor that may indirectly influence their levels of happiness and depression (Figure 1).

Previous research suggests that both happiness and depression in high-risk pregnant women are influenced by individual characteristics as well as various environmental factors. Therefore, this study aims to systematically explore the different factors that may affect happiness and depression in this demographic, drawing on ecological systems theory [15].

## Methods

Ethics statement: This study was approved by the Institutional Review Board of Kyungpook National University Chilgok Hospital (2022-06-027-001) and Kyungpook National University (2022-0357). Informed consent was obtained from the participants.

### Study design

This correlational study investigated factors influencing happiness and depression among high-risk pregnant women based on ecological systems theory. The description of this study was prepared in accordance with the STROBE reporting guidelines (https://www.strobe-statement.org/).

### Sample and sampling

The inclusion criteria were as follows: (1) being 19 years of age or older; (2) being diagnosed with a high-risk pregnancy (i.e., preterm labor, preterm premature rupture of membranes, incompetent internal os of the cervix, polyhydramnios or oligohydramnios, intrauterine growth restriction, gestational hypertension, gestational diabetes, placenta previa, multiple pregnancy, obstetrical hemorrhage, pre-existing hypertension and diabetes, history of stillbirth or preterm birth before 34 weeks, etc.) and receiving outpatient or inpatient care; (3) being at 20 to 37 weeks of gestation; (4) being married or in a common-law partnership; (5) fully understanding the purpose of the study and voluntarily providing written or online consent to participate; and (6) being able to understand and complete the questionnaire. The exclusion criteria were as follows: (1) having critical conditions, such as placental abruption, seizure, stillbirth, uterine rupture, and embolism, or being expected to have an emergency delivery; (2) having difficulty in reading and understanding Korean; (3) having the status of a married migrant or foreign woman; and (4) being diagnosed with mental illness and taking related medications.

The sample size was calculated using G*Power 3.1. Based on a median effect size of 0.15, a significance level of 0.05, and statistical power of 0.80, the number of respondents needed for the regression analysis with 13 predictor variables was 131. Considering a dropout rate of 15% [22], convenience sampling was conducted to reach approximately 154 respondents, and 153 ultimately completed the survey. After excluding one insincere response, data from 152 participants (134 face-to-face, 18 online) were used for the final analysis.

Participants were recruited both face-to-face and online to maximize the size of our cohort. For face-to-face recruitment, announcements were posted in the obstetrics outpatient departments and wards of Kyungpook National University Chilgok Hospital, as well as in two local women’s hospitals in Daegu, Korea. Research assistants were available on site to address any questions regarding participation. For online recruitment, announcements were posted on *Momsholic Baby*, the largest maternity internet community in Korea, which is used by many high-risk pregnant women seeking information and sharing their experiences.

### Measurements

Happiness was measured using the Concise Measure of Subjective Well-being, developed by Suh and Koo [10]. This tool consists of three subdomains (i.e., satisfaction, negative emotion, positive emotion) and contains nine items scored using a 7-point Likert scale (1 to 7), with higher scores (possible score range, –15 to 39) indicating higher levels of subjective well-being (i.e., happiness). The Cronbach’s alpha for reliability was .88 at the time of development [10] and .86 in this study.

Depression was measured using the Edinburgh Postnatal Depression Scale (EPDS) developed by Cox et al. [23] and translated into Korean by Han et al. [24]. Although it was developed to measure postpartum depression, the EPDS has been widely used to measure prenatal depression considering its validity and reliability. This tool contains 10 items scored using a 4-point Likert scale (0 to 3), with higher scores (possible score range, 0 to 30) indicating more severe depression. Cronbach’s alpha was .85 in the initial study of the Korean version [24] and .83 in this study.

#### Individual system level

Individual system variables consisted of pregnancy stress and mindfulness.

Pregnancy stress was assessed using the 23-item scale developed by Kim and Chung [25]. The items were scored using a 4-point Likert scale (1 to 4), with higher scores (possible range, 23 to 92) indicating higher pregnancy stress. Cronbach’s alpha was .85 at the time of development [25] and .86 in this study.

Mindfulness was measured using the mindfulness scale developed and validated by Park [26]. This 20-item tool is scored using a 5-point Likert scale (1 to 5), with higher scores (possible range, 20 to 100) indicating a higher degree of mindfulness. Cronbach’s alpha was .88 at the time of development [26] and .92 in this study.

#### Microsystem level

In the microsystem, the fetal environment (maternal-fetal interaction), family environment (marital intimacy and satisfaction with home economic condition), surrounding environment (social support), and hospital environment (satisfaction with medical services) were measured. For maternal-fetal interaction, the 10 items of the maternal-fetal interaction subscale, as part of a maternal identity tool developed by Kim and Hong [27] were used. This scale is scored using a 4-point Likert scale (1 to 4), with higher scores (possible range, 10 to 40) indicating better maternal-fetal interaction. Cronbach’s alpha was .86 at the time of development [27] and .87 in this study.

Marital intimacy was measured using the Marital Intimacy Questionnaire, which was developed by Waring and Reddon [28] and modified and supplemented by Kim [29]. Its eight items are scored on a 5-point Likert scale (1 to 4), with higher scores (possible range, 8 to 32) indicating higher marital intimacy. Cronbach’s alpha was .92 in Kim’s study [29] and .86 in this study.

Social support was assessed using a 12-item tool developed by Zimet et al. [30] and translated by Shin and Lee [31], to measure the degree of perceived support from family, friends, and significant others. Scored on a 5-point Likert scale (1 to 5), higher scores (possible range, 12 to 60) indicate higher support. Cronbach’s alpha was .89 in the study of Shin and Lee [31] and .90 in this study.

Satisfaction with home economic condition and satisfaction with medical services were each measured using one item scored on a 5-point Likert scale (1 to 5) developed by the researcher. Higher scores (possible range, 1 to 5) indicate higher satisfaction.

#### Mesosystem level

Paternal-fetal attachment was measured using the six items of the paternal-fetal attachment tool developed by Noh and Yeom [21]. Scored on a 5-point Likert scale (1 to 5), higher scores (possible range, 6 to 30) indicate better paternal behavior as observed by the pregnant woman. Cronbach’s alpha was .89 at the time of development [21] and .90 in this study.

#### Exosystem level

Community services for high-risk pregnant women were measured using two items developed by the researcher. Scored on a 5-point Likert scale (1 to 5) higher scores (possible range, 2 to 10) indicate a positive perception of community services.

### Data collection

This study was conducted from July 29 to November 28, 2022. Participants recruited from hospital sites were offered the choice of completing either a paper or an online survey. Women who opted to participate received a packet containing the paper questionnaires and a consent form, which they placed in a sealed envelope for submission. Those who selected the online option were provided with a QR code that directed them to an information sheet, an online consent form, eligibility screening questions, and the questionnaire. Participants recruited from the online community accessed the survey through a QR code announced in the posting. The research team’s contact information was made available for any inquiries about the study. The online system checked the IP address and cookies to prevent multiple entries by the same participant. Upon completing the survey, participants received a small gift valued at 8 US dollars.

### Data analysis

Data were analyzed using IBM SPSS ver. 25.0 (IBM Corp., Armonk, NY, USA). Descriptive statistics were employed to examine the general and obstetric characteristics of the participants. The individual system, microsystem, mesosystem, and exosystem variables, along with levels of happiness and depression among participants, were assessed using means and standard deviations. The t-test and one-way analysis of variance were used to explore differences in happiness and depression based on participants’ general and obstetric characteristics. Pearson correlation coefficients were calculated to determine the relationships between variables. Differences in key variables between groups receiving outpatient and inpatient care at the time of the survey were analyzed using the t-test. Hierarchical regression analysis was utilized to identify factors influencing happiness and depression among high-risk pregnant women.

## Results

The average age of the participants was 33.68±4.20 years, with 42.1% being 35 years or older. A majority, 63.6%, were experiencing their first pregnancy. About 65% of the pregnancies were planned, while 18.4% of the participants had undergone fertility treatments. Most of the participants (70.4%) had been diagnosed with at least one high-risk condition during their current pregnancy. The most frequent high-risk diagnosis was preterm labor, affecting 36.8% of the participants, followed by gestational diabetes at 33.6%. Over half of the women (52%) reported at least one hospitalization, and 9.2% had been hospitalized two or more times. At the time of the survey, approximately 45% of the participants were receiving inpatient care (Table 1).

The main dependent variable, happiness, had a relatively high mean score of 27.72±7.78. Within the subdomains of happiness, both satisfaction and positive emotion scored above the midpoint of the possible range, with mean scores of 16.01±2.97 and 15.30±3.28, respectively. In contrast, negative emotion registered a lower mean score of 10.59±3.53. Depression also recorded a fairly high mean score of 9.28±4.81. Approximately 51% of participants were at risk of depression, scoring 10 or higher. Regarding the individual system, pregnancy stress was somewhat high, averaging 51.92±10.05, while mindfulness was quite high at 84.63±10.96. The mean scores for independent variables in other systems are summarized in Table 2.

Comparisons of high-risk pregnancies categorized according to whether participants were receiving inpatient and outpatient care at the time of the survey are presented in Table 2. Our analysis revealed that the pregnancy stress score was significantly higher in the inpatient group than in the outpatient group. Furthermore, significant differences were observed in the dependent variables between the two groups: high-risk pregnant women who were hospitalized exhibited significantly lower happiness levels and higher depression levels than those who were not hospitalized.

Significant differences in happiness and depression were observed based on the general and obstetric characteristics of the participants, including maternal education level, average monthly family income, previous hospitalization experience, and current medical status at the time of the survey (Table 1). The happiness and depression levels of high-risk pregnant women showed significant correlations with the variables across all four systems. However, their depression levels did not significantly correlate with their satisfaction with community services in the exosystem (Table 3).

The variables of each system were entered into the hierarchical regression models in order, the results of which are outlined in Table 4. In the first model, pregnancy stress and mindfulness emerged as significant factors influencing happiness and depression among high-risk pregnant women, explaining 25.0% and 50.3% of the variance, respectively. In the second model, which included microsystem level variables, pregnancy stress and mindfulness continued to be significant. Additionally, high-risk pregnant women who were receiving outpatient care at the time of the survey reported higher levels of happiness (β=.26, *p*=.019). Furthermore, significant impacts on happiness were observed from maternal-fetal interaction (β=.16, *p*=.027), marital intimacy (β=.33, *p*<.001), and social support (β=.15, *p*=.022). Significant predictors of depression in this group included pregnancy stress (β=.32, *p*<.001), mindfulness (β=−.34, *p*<.001), and marital intimacy (β=−.22, *p*=.002). In the third model, neither maternal-fetal interaction nor paternal-fetal attachment in the mesosystem significantly predicted happiness. The final model revealed that marital intimacy (β=.31, *p*<.001), current outpatient medical status (β=.28, *p*=.013), mindfulness (β=.19, *p*=.013), pregnancy stress (β=−.15, *p*=.037), and social support (β=.15, *p*=.032) significantly affected the happiness of high-risk pregnant women, in descending order of impact, accounting for 51.2% of the variance. Depression was significantly influenced by mindfulness (β=−.34, *p*<.001), pregnancy stress (β=.32, *p*<.001), and marital intimacy (β=−.25, *p*=.001), which together explained 55.5% of the variance.

## Discussion

This cross-sectional survey based on ecological systems theory systematically identified factors that influence the happiness and depression of high-risk pregnant women in Korea. Our findings underscore the significance of factors within the individual system and microsystem, particularly those related to family and the immediate environment.

The happiness score in this study (20.72) was slightly higher than the average score of 19.51 for Korean adult women, as reported during the development of the tool [32]. However, it was similar to the score of 20.99 reported in low-risk Korean pregnant women using the same tool [12]. Regarding the subdomains, the negative emotion score for high-risk pregnant women in this study (10.59) was slightly higher than the score of 9.65 reported in low-risk Korean pregnant women [12]. Nevertheless, levels of life satisfaction and positive emotions were similar. These findings suggest that high-risk pregnant women have comparable levels of happiness to those of non-pregnant women, which may be related to satisfaction and positive emotions.

Conversely, high-risk pregnant women had an average depression score of 9.28, with 50.7% scoring ≥10, indicating concern. Research indicates that a cutoff of 10 points on the EPDS during the second and third trimesters yields good sensitivity, specificity, and positive predictive value for detecting depression [33]. In a prospective cohort study involving Korean pregnant women, the prevalence of prenatal depression ranged from 10.5% to 21.5%, and postpartum depression from 22.4% to 32.8%, based on a cutoff score of ≥9/10. Prenatal depression was identified as one of the strongest factors influencing postpartum depression [5]. Our findings align with those from a systematic review on psychosocial stress in high-risk pregnancies, which reported depression rates in high-risk pregnant women as high as 58% [34]. Therefore, it is crucial to manage prenatal depression in high-risk pregnant women experiencing significant stress.

Pregnancy stress was identified as a key variable that significantly influences happiness and depression in high-risk pregnant women. Research indicates that stress stemming from the physical and psychological changes during pregnancy adversely affects maternal happiness [12]. This aligns with findings that increased perceived stress correlates with a higher incidence of depressive symptoms during pregnancy [16]. Specifically, perceived stress during pregnancy has been strongly associated with preterm birth [35]. Pregnant women with pre-existing high-risk factors may experience elevated levels of pregnancy stress, potentially leading to depression and preterm birth, which in turn adversely affects fetal health. Our findings indicate that pregnancy stress levels are significantly higher among hospitalized high-risk pregnant women compared to those receiving outpatient care, highlighting the increased vulnerability of hospitalized high-risk pregnant women to prenatal depression. It has been reported that greater social support can mitigate the effects of maternal stress on prenatal depression [16]. Therefore, assessing stress levels, prenatal depressive symptoms, and social support in women diagnosed with high-risk pregnancies is crucial. Implementing interventions that strengthen social support for high-risk pregnant women, particularly those who are hospitalized, could be an effective strategy to not only boost their happiness but also prevent depressive symptoms.

The current study identified mindfulness as a significant individual factor that increases happiness and decreases depression in high-risk pregnant women. A systematic review examining the effects of mindfulness interventions on maternal well-being demonstrated that these interventions decrease anxiety, depression, and negative emotions while increasing self-compassion [18]. Reducing negative emotions and increasing compassion toward oneself can help increase happiness in pregnant women. Another systematic review found that mobile health (mHealth) had an effect on maternal psychosocial health and may be beneficial for high-risk pregnant women who are vulnerable to psychological problems [36]. High-risk pregnant women hospitalized for preterm labor were found to rely heavily on their smartphones [37]. Moreover, an international study showed that a short-term mHealth-based mindfulness program effectively reduced anxiety among hospitalized high-risk pregnant women [38]. Therefore, implementing mHealth mindfulness programs for high-risk pregnant women, especially those who are hospitalized, may help alleviate anxiety and depression while boosting their happiness.

At the microsystem level, the second model revealed that maternal-fetal interaction increased the happiness of high-risk pregnant women. Similarly, previous research has shown that maternal-fetal bonding can moderate the effects of pregnancy-related stress on psychological well-being [39]. In this study, interacting with the fetus may have helped mitigate the adverse impacts of stress on the happiness of high-risk pregnant women. It has been reported that high-risk pregnancies, particularly those requiring hospitalization, may pose a risk to the development of maternal-fetal attachment [40]. However, family support has been found to partially mediate the effects of anxiety and depression on maternal-fetal attachment among hospitalized women with high-risk pregnancies [41]. Therefore, when developing programs to enhance the happiness of high-risk pregnant women, it is crucial to focus on strengthening family support.

Meanwhile, marital intimacy was found to be one of the most important factors affecting both happiness and depression in high-risk pregnant women. A study on low-risk Korean pregnant women found that marital intimacy was a stronger predictor of happiness than individual factors, such as personality and body image [12]. Furthermore, a review focused on the well-being of high-risk pregnant women suggested that interpersonal relationships precede well-being in this group [42]. These findings align with our own results showing that a healthy, intimate marital relationship was associated with higher happiness and lower depression levels. Notably, women whose spouses participated in prenatal programs reported a significant improvement in marital intimacy compared to those whose spouses did not participate [43]. This underscores the importance of involving spouses in interventions designed to improve marital intimacy among high-risk pregnant women.

Social and emotional support from family, spouses, coworkers, neighbors, healthcare providers, and others can enhance the well-being of pregnant women [42]. Our study identified social support as a microsystem factor that positively influenced the happiness of high-risk pregnant women; however, it did not significantly affect depression levels. This finding contrasts with a previous study, which suggested that having supportive individuals during pregnancy could prevent postpartum depression, particularly among those already suffering from depression [44]. The discrepancy in findings may be due to differences in the study populations, as approximately 45% of our participants were hospitalized. In our research, social support was defined as assistance received from family, friends, and significant others. Given the challenges of hospital visitation during the study period due to COVID-19, this support likely had a minimal impact on the high levels of depression reported by hospitalized high-risk pregnant women. Meanwhile, social media is emerging as a potential support system [45]. Leveraging social media to enhance social support for high-risk pregnant women with limited mobility, such as those experiencing preterm labor or premature rupture of membranes, could prove to be an effective strategy.

The current study found that women’s perception of paternal-fetal attachment, considered as the mesosystem, did not influence happiness or depression in high-risk pregnant women. However, other studies have demonstrated that a paternal-fetal attachment program enhanced marital satisfaction among couples experiencing low-risk pregnancies [46]. This suggests that fostering paternal-fetal attachment could potentially increase perceived satisfaction and intimacy between spouses during pregnancy. The study also identified marital intimacy as a key factor influencing happiness and depression in high-risk pregnant women. Consequently, promoting paternal-fetal attachment may be an effective strategy to promote marital intimacy in these high-risk situations. Furthermore, our findings indicated that the exosystem variable did not impact either happiness or depression in high-risk pregnant women. This contrasts with findings from a study on low-risk pregnant women in Korea, where community services within the exosystem played a significant role [12]. This discrepancy underscores the need for tailored support. In Korea, support for high-risk pregnant women primarily covers medical expenses through the Ministry of Health and Welfare [47]. Maternal-fetal intensive care units, which are the main care centers for these women, focus predominantly on medical treatment and offer minimal psychological support [48]. Most community services and programs are designed with low-risk pregnancies in mind, such as in-person programs. There is a clear need for community pregnancy programs, such as *taegyo*, meditation programs, and others, to be accessible to high-risk pregnant women. In this context, utilizing mHealth could be a viable strategy. Vulnerable groups, including hospitalized high-risk pregnant women, have experienced benefits from using mHealth [36, 38].

A limitation of this study is that most participants were recruited from a single city; therefore, our results should be generalized with caution. Additionally, as the present study incorporated only certain factors from the ecological systems theory, future research should consider including other relevant factors.

In conclusion, the findings of this study indicate that happiness and depression are not polar opposites in high-risk pregnant women. There is a need for interventions aimed at reducing depression, particularly in those who are hospitalized, as well as initiatives to enhance happiness. Factors such as pregnancy stress, mindfulness, and marital intimacy commonly affect both happiness and depression in these women, though various environmental system factors may also play a role. Efforts to strengthen these common elements (mHealth utilization, greater spousal involvement, etc.), as well as the development of programs that consider additional environmental influences (i.e., hospitalization, social support), are needed. Additionally, there is a need to improve continuing education for nurses who care for high-risk pregnant women, ensuring that they can provide care that incorporates these findings.

## Figures and Tables

**Table 1.** Differences in happiness and depression according to general and obstetric characteristics (N=152)

Characteristic	Categories	n (%) or Mean±SD	Happiness		Depression	
			Mean±SD	t/F/Z (*p*)	Mean±SD	t/F/Z (*p*)
Age (year)		33.68±4.20				
	<35	88 (57.9)	19.75±7.90	–1.81 (.072)	9.64±4.91	1.08 (.281)
	≥35	64 (42.1)	22.05±7.46		8.78±4.68	
Education level	<University	60 (39.4)	19.03±8.28	–2.18 (.031)	10.65±4.95	2.91 (.004)
	≥University	92 (60.6)	21.82±7.27		8.38±4.53	
Religion	Yes	55 (36.2)	21.39±7.45	1.43 (.156)	10.27±5.28	–1.94 (.054)
	No	97 (63.8)	19.53±8.27		8.71±4.46	
Working status	Working	34 (22.4)	21.65±6.57	0.99 (.374)	8.21±5.00	1.43 (.243)
	On leave	50 (32.9)	21.42±7.42		9.16±4.95	
	Unemployed	68 (44.7)	19.74±8.55		9.90±4.58	
Monthly income (KRW)	≤4 million	80 (52.6)	19.46±7.80	–2.12 (.036)	10.30±5.03	2.83 (.005)
	> 4 million	72 (47.4)	22.11±7.56		8.14±4.32	
Marital status^†^	Married	148 (97.4)	20.78±7.66	–0.37 (.360)	9.20±4.83	–1.38 (.089)
	Common-law partnership	4 (2.6)	18.50±12.61		12.25±3.30	
Length of marriage (month)		46.01±33.66				
	<48	90 (59.2)	20.72±7.81	–0.01 (.992)	9.09±4.96	0.58 (.565)
	≥48	62 (40.8)	20.71±7.79		9.55±4.61	
Number of children (N=151)	0	96 (63.6)	21.25±7.38	1.17 (.244)	9.06±4.97	–0.66 (.512)
	≥1	55 (36.4)	19.71±8.45		9.60±4.58	
Gestational age (week)	20-27	45 (29.6)	21.93±7.71	1.25 (.212)	8.91±4.57	–0.60 (.546)
	28-36	107 (70.4)	20.21±7.78		9.43±4.93	
Planned pregnancy (N=151)	Yes	99 (65.6)	21.28±7.01	1.23 (.224)	9.00±4.59	–1.07 (.285)
	No	52 (34.4)	19.52±9.05		9.88±5.22	
Infertility treatment	Yes	28 (18.4)	21.32±7.94	0.45 (.650)	8.93±4.79	–0.42 (.674)
	No	124 (81.6)	20.58±7.76		9.35±4.83	
Number of diagnoses^‡^	1	107 (70.4)	20.85±8.00	0.33 (.746)	8.98±4.95	–1.17 (.245)
	≥2	45 (29.6)	20.40±7.30		9.98±4.43	
Hospitalization experience	Yes	79 (52.0)	19.52±7.60	–2.00 (.048)	10.65±4.27	3.79 (<.001)
	No	73 (48.0)	22.01±7.81		7.79±4.95	
Medical status at the time of survey	Inpatient	69 (45.4)	18.97±7.52	2.57 (.011)	10.77±4.40	–3.62 (<.001)
	Outpatient	83 (54.6)	22.17±7.73		8.04±4.81	
Recruitment method^†^	Online	18 (11.8)	21.67±5.96	–0.38 (.706)	8.89±4.83	–0.03 (.973)
	Offline	134 (88.2)	20.59±8.00		9.33±4.83	

KRW: Korean won. One million KRW is approximately 800 US dollars.

† Mann-Whitney U test.

‡ Diagnosis: preterm labor (36.8%), incompetent internal os of the cervix (10.5%), gestational hypertension (9.9%), gestational diabetes (33.6%), placenta previa (6.6%), oligohydramnios or polyhydramnios (4.6%), intrauterine growth restriction (7.9%), multiple pregnancy (13.8%), causes of obstetrical hemorrhage (4.6%), preeclampsia (0.7%), others (uterine myoma, overt diabetes mellitus, hypothyroidism, aplastic anemia, femoroacetabular impingement, twin to twin infusion syndrome, rare and intractable disorder; 6.6%).

**Table 2.** Levels of individual system, microsystem, mesosystem, and exosystem factors, as well as happiness and depression (N=152)

Concept	Variable	Total score Mean±SD	Min	Max	Possible range	Outpatient (n=83) Mean±SD	Inpatient (n=69) Mean±SD	t	*p*
Individual									
	Pregnancy stress	51.92±10.05	29	79	23 to 92	49.71±9.91	54.58±9.63	–3.05	.003
	Mindfulness	84.63±10.96	53	100	20 to 100	85.72±11.04	83.32±10.80	1.35	.179
Microsystem									
	Maternal-fetal interaction	28.64±5.95	12	40	10 to 40	29.04±6.18	28.16±5.68	0.90	.368
	Marital intimacy	25.27±4.37	13	32	8 to 32	24.87±4.51	25.75±4.17	–1.25	.214
	Satisfaction with home economic condition	3.39±0.82	1	5	1 to 5	3.47±0.86	3.29±0.77	1.35	.180
	Social support	46.43±8.31	21	60	12 to 60	47.42±7.83	45.23±8.76	1.63	.106
	Satisfaction with medical services	4.18±0.67	2	5	1 to 5	4.19±0.69	4.16±0.66	0.30	.762
Mesosystem									
	Paternal-fetal attachment	21.73±5.56	6	30	6 to 30	21.77±5.49	21.68±5.69	0.10	.921
Exosystem									
	Community services	5.58±1.72	2	10	2 to 10	5.82±1.65	5.29±1.77	1.90	.059
	Happiness	27.72±7.78	2	39	–15 to 39	22.17±7.73	18.97±7.52	2.57	.011
	Satisfaction	16.01±2.97	8	21	3–21	16.42±2.94	15.52±2.94	1.88	.062
	Positive emotion	15.30±3.28	6	21	3 to 21	15.86±3.03	14.62±3.46	2.34	.021
	Negative emotion	10.59±3.53	3	17	3 to 21	10.11±3.43	11.17±3.59	–1.87	.064
	Depression^†^	9.28±4.81	0	23	0 to 30	8.04±4.81	10.77±4.40	–3.62	<.001

† There were 77 (50.7%) participants ≥10 and 75 (49.3%) <10.

**Table 3.** Relationships among individual system, microsystem, mesosystem, and exosystem factors, as well as happiness and depression (N=152)

Variable	STR	MF	MFI	INT	ECO	SS	MED	PFA	CS	HAP
STR	1									
MF	–.57 (<.001)	1								
MFI	–.10 (.243)	.05 (.524)	1							
INT	–.20 (.014)	.28 (.001)	.44 (<.001)	1						
ECO	–.28 (<.001)	.24 (.003)	.25 (.002)	.41 (<.001)	1					
SS	–.22 (.007)	.26 (.001)	.42 (<.001)	.37 (<.001)	.32 (<.001)	1				
MED	–.24 (.003)	.25 (.002)	.20 (.011)	.15 (.057)	.23 (.004)	.15 (.059)	1			
PFA	–.17 (.034)	.19 (.020)	.55 (<.001)	.59 (<.001)	.41 (<.001)	.34 (<.001)	.13 (.102)	1		
CS	–.03 (.750)	–.02 (.835)	.37 (<.001)	.16 (.043)	.24 (.003)	.30 (<.001)	.22 (.007)	.33 (<.001)	1	
HAP	–.43 (<.001)	.46 (<.001)	.42 (<.001)	.56 (<.001)	.39 (<.001)	.47 (<.001)	.26 (.001)	.46 (<.001)	.22 (.006)	1
DEP	.62 (<.001)	–.62 (<.001)	–.22 (.006)	–.39 (<.001)	–.31 (<.001)	–.21 (.008)	–.26 (.001)	–.25 (.002)	–.10 (.239)	–.65 (<.001)

CS: Community services; DEP: depression; ECO: satisfaction with home economic condition; HAP: happiness; INT: marital intimacy; MED: satisfaction with medical services; MF: mindfulness; MFI: maternal-fetal interaction; PFA: paternal-fetal attachment; SS: social support; STR: pregnancy stress.

**Table 4.** Factors influencing happiness and depression (N=152)

Concept	Categories/Variables		Happiness, β (*p*)				Depression, β (*p*)			
			Model 1	Model 2	Model 3	Model 4	Model 1	Model 2	Model 3	Model 4
Individual system	Education level (≥university=1)		.06 (.404)	.01 (.815)	.01 (.835)	.01 (.835)	–.08 (.181)	–.06 (.283)	–.06 (.270)	-.06 (.272)
	Hospitalization experience (no=1)		–.10 (.476)	–.15 (.172)	–.17 (.137)	–.18 (.119)	–.13 (.245)	–.10 (.362)	–.11 (.289)	-.11 (.300)
	Medical status at the time of survey (outpatient=1)		.19 (.156)	.26 (.019)^†^	.27 (.015)^†^	.28 (.013)^†^	–.04 (.729)	–.09 (.401)	–.07 (.488)	-.07 (.493)
	Pregnancy stress		–.22 (.013)^†^	–.15 (.041)^†^	–.15 (.041)^†^	–.15 (.037)^†^	.35 (<.001)^†^	.32 (<.001)^†^	.32 (<.001)^†^	.32 (<.001)^†^
	Mindfulness		.31 (.001)^†^	.19 (.013)^†^	.18 (.015)^†^	.19 (.013)^†^	–.38 (<.001)^†^	–.34 (<.001)^†^	–.34 (<.001)^†^	-.34 (<.001)^†^
Microsystem	Fetus	Maternal-fetal interaction		.16 (.027)^†^	.13 (.083)	.12 (.103)		–.08 (.217)	-.11 (.123)	-.11 (.129)
	Family	Monthly income≥401 (×10,000 KRW)		.04 (.500)	.04 (.501)	.04 (.553)		–.08 (.187)	-.08 (.186)	-.08 (.192)
		Marital intimacy		.33 (<.001)^†^	.30 (<.001)^†^	.31 (<.001)^†^		–.22 (.002)^†^	-.25 (.001)^†^	-.25 (.001)^†^
		Satisfaction with home economic condition		.04 (.523)	.03 (.671)	.03 (.700)		–.01 (.874)	-.03 (.701)	-.03 (.702)
	Surroundings	Social support		.15 (.022)^†^	.15 (.023)^†^	.15 (.032)^†^		.10 (.108)	.10 (.111)	.10 (.118)
	Hospital	Satisfaction with medical services		.07 (.239)	.08 (.216)	.07 (.262)		–.06 (.312)	-.06 (.350)	-.06 (.359)
Mesosystem	Paternal-fetal attachment				.08 (.336)	.07 (.382)			.08 (.273)	.08 (.280)
Exosystem	Community services					.04 (.596)				.00 (.995)
R^2^ (adjusted R^2^)			.275 (.250)	.550 (.514)	.553 (.514)	.554 (.512)	.519 (.503)	.590 (.558)	.593 (.558)	.593 (.555)
△R^2^			.275	.275	.003	.001	.519	.071	.004	.000
F			11.07	15.54	14.31	13.16	31.52	18.30	16.90	15.49
*p*			<.001	<.001	<.001	<.001	<.001	<.001	<.001	<.001

KRW: Korean won. One million KRW is approximately 800 US dollars.

† Statistically significant results.
